# Transcranial Current Stimulation as a Tool of Neuromodulation of Cognitive Functions in Parkinson’s Disease

**DOI:** 10.3389/fnins.2022.781488

**Published:** 2022-07-12

**Authors:** Ivan V. Brak, Elena Filimonova, Oleg Zakhariya, Rustam Khasanov, Ivan Stepanyan

**Affiliations:** ^1^Laboratory of Comprehensive Problems of Risk Assessment to Population and Workers’ Health, Federal State Budgetary Scientific Institution “Izmerov Research Institute of Occupational Health”, Moscow, Russia; ^2^“Engiwiki” Scientific and Engineering Projects Laboratory, Department of Information Technologies, Novosibirsk State University, Novosibirsk, Russia; ^3^Federal Neurosurgical Center, Novosibirsk, Russia; ^4^Faculty of Philosophy, Lomonosov Moscow State University, Moscow, Russia; ^5^Independent Researcher, Novosibirsk, Russia; ^6^Peoples’ Friendship University of Russia (RUDN University), Moscow, Russia; ^7^Mechanical Engineering Research Institute of the Russian Academy of Sciences, Moscow, Russia

**Keywords:** Parkinson disease (PD), mild cognitive impairment (MCI), transcranial magnetic stimulation (TMS), transcranial alternating current stimulation (tACS), transcranial direct current stimulation (HD-tDCS)

## Abstract

Decrease in cognitive function is one of the most common causes of poor life quality and early disability in patients with Parkinson’s disease (PD). Existing methods of treatment are aimed at both correction of motor and non-motor symptoms. Methods of adjuvant therapy (or complementary therapy) for maintaining cognitive functions in patients with PD are of interest. A promising subject of research in this regard is the method of transcranial electric current stimulation (tES). Here we reviewed the current understanding of the pathogenesis of cognitive impairment in PD and of the effects of transcranial direct current stimulation and transcranial alternating current stimulation on the cognitive function of patients with PD-MCI (Parkinson’s Disease–Mild Cognitive Impairment).

## Introduction

Parkinson’s disease (PD) is a neurodegenerative disease of unknown etiology with a steadily progressive course leading to severe disability (Pringsheim et al., 2014). PD affects about 1% of the population over 60 years of age and ranks second after Alzheimer’s disease in the structure of neurodegenerative pathologies. The most dominant in the clinical picture of PD are motor symptoms such as tremor, rigidity, bradykinesia, and postural instability. However, non-motor symptoms (i.e., cognitive decline, affective disorders, and sleep disturbances) have also been shown to be one of the main causes of reduced quality of life in patients with PD (Aarsland et al., 2017). These symptoms appear early in the disease, increase over time, and reflect the progression of Parkinson’s disease more accurately than motor symptoms (van der Heeden et al., 2014).

Recent epidemiological and clinical studies suggest that “mild cognitive impairment” (MCI) may be a complex typical of the early stages of PD (Manenti et al., 2016). Patients with Parkinson’s Disease–Mild Cognitive Impairment (PD-MCI) show changes in various aspects of cognitive activity, such as attention, visuospatial perception, executive functions, memory (Sauerbier et al., 2016), but in most cases these factors do not significantly affect the daily activities of patients. MCI is present in approximately 25% of cases of idiopathic parkinsonism and is a risk factor for dementia.

Nevertheless, all existing methods of PD treatment (dopaminergic replacement therapy, deep brain stimulation) are mainly aimed at correcting motor symptoms and practically do not affect the severity of non-motor symptoms. Thus, it is necessary to develop and implement adjuvant therapy aimed at maintaining cognitive functions in patients with PD.

Over the past two decades, it has been shown that transcranial current stimulation (tCS) of the brain (TTS) has a positive effect on cognitive activity in healthy people, as well as a therapeutic effect in mental (depression, schizophrenia) and neurodegenerative (Alzheimer’s and Parkinson’s) disorders (Kuo et al., 2014). tCS is a non-invasive, potentially portable, proven method of brain stimulation with minimal risk of side effects. This allows us to consider TCS as one of the methods of adjuvant therapy, which can theoretically be used to control mild cognitive impairment in PD.

There are a number of successful studies showing a positive effect of transcranial direct current stimulation (tDCS) on the cognitive functions of patients with PD, both in single (Boggio et al., 2006) and multi-session interventions (Manenti et al., 2016).

Transcranial alternating current stimulation (tACS) is a relatively new neuromodulation technology aimed at changing the functional activity of specialized brain networks using modulation through the mechanism of involving endogenous brain oscillations with an externally set frequency generated in the EEG ranges (from 0.1 to 100 Hz) (Helfrich et al., 2016). Thus, tACS modulates the natural oscillatory activity of the cortex (Schutter, 2014).

One target for tACS that is of interest for research in patients with MCI is the mean frontal theta rhythm (FMT): its amplitude is thought to be positively correlated with cognitive areas such as executive functions and working memory (Cavanagh and Frank, 2014). Moreover, studies in patients with Parkinson’s disease have shown a decrease in FMT amplitude due to deterioration in cognitive control functions (Singh et al., 2018).

Thus, one of the causes of cognitive impairment in PD is a decrease in the activation of neurons in the prefrontal cortex due to a decrease in dopamine concentration (Kehagia et al., 2013). On the other hand, it is believed that a positive effect from the use of TCS techniques is achieved by modulating neuroplasticity processes (Zimerman and Hummel, 2010). In addition, transcranial electrical stimulation has been shown to have neuroprotective properties, reducing the severity of oxidative stress in dopaminergic neurons (Lu et al., 2015).

Significant progress in the methods of transcranial magnetic stimulation should be noted; new stimulation protocols are being developed to increase its effectiveness. Of particular interest are Theta-burst stimulation (iTBS), transcranial random noise stimulation (tRNS) (Monastero et al., 2020) and amplitude modulated transcranial alternating current stimulation (AM-tACS) in the treatment of Parkinson’s disease. however, they require a separate systematic review.

As part of the review, we are investigating two methods of transcranial stimulation: AC and DC stimulation.

## Pathogenesis of Cognitive Impairment in Parkinson’s Disease

Cognitive impairment is one of the most common non-motor manifestations of Parkinson’s disease (Draoui et al., 2020): as a rule, it is already present at the time of diagnosis, and the total prevalence of PD-associated dementia reaches 75–90% with a disease duration of more than 10 years (Aarsland and Kurz, 2010). Cognitive impairment negatively affects the daily activity of PD patients (Rosenthal et al., 2010) and increases the mortality rate and the risk of developing other diseases (Levy et al., 2002).

The pathophysiological mechanisms underlying the PD-associated dementia have not been sufficiently studied to date. As a rule, cognitive deficiency in PD is usually associated with neurochemical shifts in the work of dopaminergic, cholinergic and other mediator systems of the central nervous system. Lewy bodies in the neurons of the brain’s limbic system, amyloid plaques, as well as cerebrovascular changes (Gratwicke et al., 2015; Hanagasi et al., 2017) act as a neuropathological substrate during these shifts.

**FIGURE 1:**
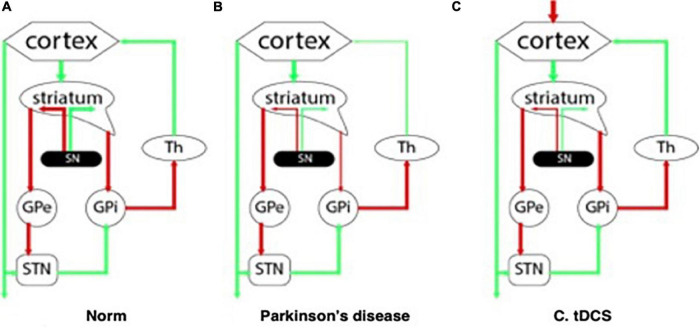
Interaction between the structures of the central nervous system: **(A)** at normal; **(B)** with Parkinson’s disease; **(C)** in the pathogenetic therapy of Parkinson’s disease using tDCS.

The clinical cognitive profile of patients with PD-associated dementia reflects the damage to the subcortical structures and dysregulation in the “cortex – basal ganglia – thalamus” system. Degeneration of the main dopaminergic cortical terminals leads to dopaminergic deficiency and disruption of the normal functioning of neuronal ensembles in the corresponding parts of the cerebral cortex (Figures 1A,B). In this regard, with mild cognitive impairment primarily noted changes are in the executive functions (Gratwicke et al., 2015), and domains such as declarative memory, language and praxis, on the contrary, remain intact for a long time.

Executive functions are such cognitive abilities as decision making, planning, behavior in a paradigm shift and inhibition of the response to the stimulus (Dirnberger and Jahanshahi, 2013). Disruptions in the normal functioning of the executive function domain are usually observed in patients with Parkinson’s disease at the time of diagnosis (Muslimovic et al., 2005), and such disorders can be part of the prodromal period (Goldman, 2014). Changes progress along with the disease (Christopher et al., 2014), and symptoms such as decreased attention span, forgetfulness, and loss of planning and organization skills gradually increase (Bronnick et al., 2006).

It is known that prefrontal cortex (PFC) is associated with executive functions. This area of the brain actively interacts with the structures of the striatum through the dopamine-dependent corticostriate neuronal pathway (Middleton and Strick, 2000), as well as with the ventral tegmental area (VTA) of the midbrain via the mesocortical pathway (Fuster, 2015). The work of both pathways is disrupted in Parkinson’s disease due to degeneration of dopaminergic neurons of the substantia nigra and VTA with failures in the modulation of neuronal ensembles and the equilibrium between direct and indirect loops (Draoui et al., 2020). Thus, cognitive deficits in Parkinson’s disease can be explained by a decrease in neuronal activation in the prefrontal cortex due to a decrease in dopamine concentration (Kehagia et al., 2013).

However, in addition to the classic executive function dysfunction syndrome, the clinical phenotype of PD-associated dementia also includes memory, attention, and visual-spatial perception impairments (Pagonabarraga and Kulisevsky, 2012; Kehagia et al., 2013), cognitive fluctuations, and sometimes visual hallucinations (Emre, 2003). Thus, disorders in the cholinergic system contribute to the formation of cognitive deficit in PD due to degeneration of the Meinert basal nucleus, a decrease in cholinergic transmission in the cerebral cortex, which leads to a decrease in attention and learning functions (Gratwicke et al., 2015). “Dual syndrome hypothesis” (Kehagia et al., 2013) refers to the presence of two different types of cognitive impairment in PD. The first type is a fronto-striatal deficit of attention and/or executive functions, that presents mild cognitive impairment (MCI) in the form of an executive and working memory deficit, and is caused by a deficiency of the catecholaminergic system. The second type of cognitive impairment presents itself in the form of visual-spatial perception impairments, and is associated with a higher risk of developing dementia with a characteristic pattern of cognitive decline, agnosia, apraxia, and aphasia.

Based on the entire spectrum of clinical manifestations, criteria for diagnosing PD-associated dementia were developed (Emre et al., 2007), according to which diagnosis must be made based on changes in at least two cognitive domains (attention, executive functions, visual and spatial functions or memory) and at least one symptom of a behavioral disorder (apathy, daytime sleepiness, hallucinations, delirium, affective disturbances).

In addition to “dementia,” the term “mild cognitive impairment,” which reflects a more subtle decrease in cognitive function in patients compared to age-related control, and may be a prodromal state of dementia (Baiano et al., 2020), has been widely used in clinical practice in recent decades.

Mild cognitive impairment suggests changes in at least one of the five cognitive domains: long-term memory, attention and working memory, visual-spatial abilities, executive functions, and language. In addition, mild cognitive impairment is further devided into a mono- or multi-domain lesion MCI. MCI diagnosis in PD is based on the MDS (Movement Disorder Society) criteria developed in 2012 (Litvan et al., 2012).

Mild cognitive impairment is believed to be a major risk factor for the development of PD-associated dementia (Baiano et al., 2020). Thus, therapeutic interventions aimed at improving cognitive function in PD-MCI patients are potentially promising.

## Changes in Brain Oscillatory Activity in Parkinson’s Disease–Mild Cognitive Impairment

Changes in the oscillatory activity of the central nervous system in resting state in patients with Parkinson’s disease compared to healthy controls of the same age were repeatedly recorded (Tessitore et al., 2019). According to the latest data (Geraedts et al., 2018), in most studies using quantitative EEG in patients with PD, a decrease in the frequency of an individual alpha peak, combined with an increase in power in the delta range, was recorded in comparison with healthy controls. In addition, a general slowdown in the oscillatory activity of the brain is noted in PD, which correlates with the severity of cognitive deficit (Caviness et al., 2007, 2015). Currently, EEG analysis using non-linear methods is gaining momentum. There are studies on the relationship between Parkinson’s disease and various non-linear changes in EEG signals (Lee et al., 2021).

Earlier studies (Soikkeli et al., 1991) revealed a decrease in the relative amplitude of alpha and beta rhythms and an increase in the amplitude of the theta rhythm in patients with Parkinson’s disease compared to the controls. Neufeld et al. (1994) showed a decrease in the relative amplitude of EEG activity in the alpha range in patients with PD-associated dementia compared to healthy controls and patients with PD without cognitive impairment.

However, not all studies have revealed the same patterns of change in the power of oscillations. Tanaka et al. (2000) and Moazami-Goudarzi et al. (2008) showed an increase in power in all ranges in PD, while Stoffers et al. (2007) and Stanzione et al. (2011) only observed changes in individual ranges: increase in mean power δ, as well as a decrease in power β1, increase in theta and power α, decrease in power γ. Nevertheless, there seems to be a pattern that the ratio of EEG power at high frequencies to power at low frequencies, as well as the individual frequency of the alpha peak, correlate with the state of the cognitive domain (Caviness et al., 2015; Guner et al., 2017).

## Middle Frontal Theta Rhythm Changes in Parkinson’s Disease–Mild Cognitive Impairment

It is believed that oscillations of the medial frontal cortex in the theta range (the so-called middle frontal theta rhythm) characterize the course of decision-making processes (van Rijn et al., 2011). Moreover, it is one of the candidates for the role of a biophysical substrate for cognitive control (Cavanagh and Frank, 2014). It is believed that one of the sources of the FMT is the anterior cingulate cortex (ACC) (Tsujimoto et al., 2006; Womelsdorf et al., 2007). This area is related to the processes of memory involvement, attention, learning and decision making (Debener et al., 2005; Marco-Pallares et al., 2008). Other sources are considered to be the middle cingulate cortex (MCC) and the pre-supplementary motor area (preSMA), where the FMT oscillations take part in mechanisms connected to adaptive control in situations with an ambiguous outcome (Cavanagh et al., 2012).

Executive functions, attention and working memory are cognitive functions that are one way or another dependent on FMT, and their impairment is an essential part of the clinical symptoms of patients with PD-MCI (Gratwicke et al., 2015).

Studies on animal models showed that oscillatory activity in the delta range at the time of performing a cognitive task was reduced in mice with a local dopamine deficiency in MFC (medial frontal cortex) (Parker et al., 2015). In human studies, it was found that low-frequency EEG activity in healthy volunteers is positively correlated with the success of performing neuropsychological tests for executive functions [WCST (Wisconsin Card Sorting Test), TMT (Treadmill test), Stroop], while such differences could not be detected in patients with Parkinson’s disease (Parker et al., 2015). Based on this, it can be assumed that a stable supply of dopamine through the mesocortical pathways is necessary for the normal functioning of low-frequency oscillations at the level of the medial frontal cortex.

## Changes in Resting State Functional Magnetic Resonance Imaging Functional Connectivity in Parkinson’s Disease–Mild Cognitive Impairment

To date, a large number of studies have been published on changes in resting state functional magnetic resonance imaging (rs-fMRI) functional connectivity between brain regions in patients with PD and cognitive impairment. However, the heterogeneity of existing data regarding the pattern of changes in connectivity, as well as the relationship between the functioning of individual cognitive domains and the level of connectivity in their specific brain regions (Baggio et al., 2019) should be noted.

Nevertheless, in one of the first studies in this area (Tessitore et al., 2012), a decrease in rs-fMRI functional connectivity was recorded in the lower parietal cortex bilaterally and in the medial temporal cortex of the right hemisphere in patients with PD, in contrast to the control group. At the same time, indicators of the temporal cortex connectivity correlated with the successful performance on the memory evaluation tests, while the connectivity indicators in the parietal cortex correlated with the state of visual-spatial functions.

Changes in resting state rs-fMRI functional connectivity in PD-MCI patients have been most studied within the default mode network (DMN) (Baggio et al., 2019).

Hou et al. (2016) showed a decrease in rs-fMRI functional connectivity within the DMN in patients with PD compared to the control group. The level of connectivity between the anterior temporal cortex and the middle temporal gyrus correlated with attention and working memory functions, while the level of connectivity between the hippocampus and the lower frontal gyrus correlated only with the memory functions.

Lucas-Jiménez et al. (2016) showed a decrease in rs-fMRI functional connectivity between the posterior cingulate cortex (PCC) and the medial portions of the temporal cortex bilaterally. The level of connectivity between PCC and the right medial temporal cortex correlated with the state of visual and verbal memory, while the level of connectivity between PCC and the left medial temporal cortex correlated with visual recognition.

Baggio et al. (2015) revealed a decrease in rs-fMRI functional connectivity between the dorsal attention network and the right islet in patients with PD-MCI, and the changes correlated with the state of executive functions and attention. It was also shown that a registered decrease in the level of connectivity is not accompanied by structural degeneration of the brain tissue. In general, this fact can be attributed to the hypothesis about the role of dopamine imbalance in the anterior islet cortex in the etiology of attention loss and executive functions in PD (Christopher et al., 2014).

On the other hand, the same study (Baggio et al., 2015) recorded an increase in the level of connectivity between the nodes of the DMN and the posterior parietal cortex in patients with PD-MCI compared to healthy controls, which correlated with a deterioration in visual and spatial functions, as well as with the thickness of the occipital-parietal cortex. These data confirm the hypothesis that the deterioration of visual-spatial functions in Parkinson’s disease is a consequence of the parieto-occipital cortex degeneration due to sinucleinopathy, but not secondary to dopamine deficiency (Williams-Gray et al., 2009).

Gorges et al. (2015) revealed an increase in the level of connectivity within the main resting state networks (DMN, frontoparietal network, attention network) in patients with PD without cognitive impairment compared to the healthy control group. In the PD-MCI patients, its level, on the contrary, was reduced. This fact can be explained by the use of compensatory mechanisms in the relatively early stages of the development of the disease.

## Transcranial Direct Current Stimulation and Transcranial Alternating Current Stimulation: Review of the Method and Mechanisms of Action

### Transcranial Direct Current Stimulation

Transcranial direct current stimulation (tDCS) is a non-invasive brain stimulation technique used to modulate the excitability of the cortex (Ferrucci et al., 2018).

It is believed that the main effects of tDCS are achieved due to a subthreshold shift of the resting potentials of the neuronal cell membrane toward de- or hyperpolarization (depending on the direction of the current relative to the orientation of the axons) (Bindman et al., 1964).

**FIGURE 2:**
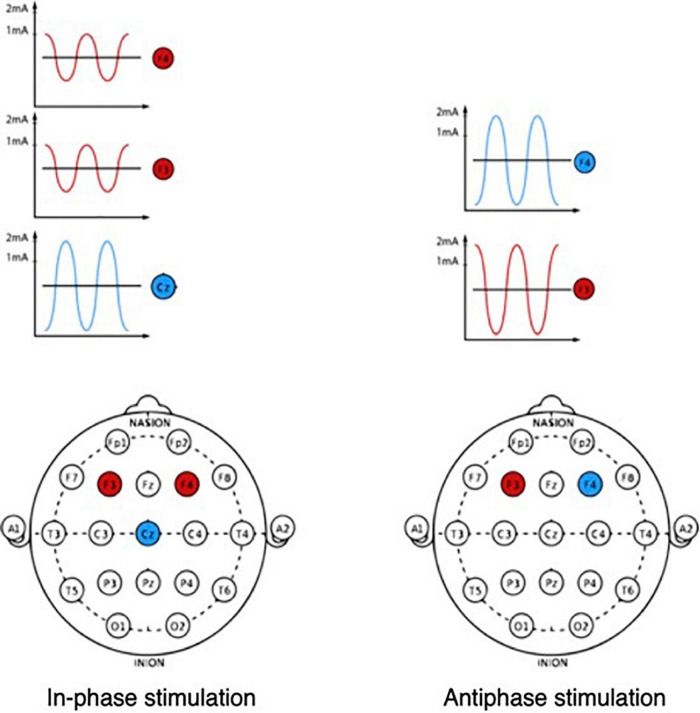
The tDCS mechanism. Shift of membrane excitability when exposed to a weak direct current.

During the tDCS procedure, a weak electric current is applied to the scalp between two electrodes: the anode and the cathode (Figure 2). The interaction between the current and nerve tissue causes a shift in membrane excitability: as a rule, nerve tissue is depolarized under the anode, and hyperpolarized under the cathode. Thus, the therapeutic potential of this method is based on the fact that local displacements of excitability in various regions of the brain can affect abnormal patterns of neuronal activity that form under various pathological conditions, including PD (Ferrucci et al., 2018), by starting processes that form the neuroplasticity and compensatory mechanisms.

It is known that anodal tDCS increases the excitability of the stimulated cortex, which is manifested in an increase in the amplitude of the motor evoked potential (MEP), while the cathodal tDCS, on the contrary, decreases cortical excitability (Nitsche and Paulus, 2000). In order to cause the described changes in excitability, stimulation lasting several seconds is sufficient (Nitsche and Paulus, 2000). An increase in stimulation time (several minutes) increases the persistence of changes that can last for more than 1 h (Nitsche and Paulus, 2001; Nitsche et al., 2003; Priori, 2003).

The mechanisms of action of tDCS (including therapeutic tDCS) have not yet been fully understood. Nevertheless, there are several hypotheses that explain the effect. For example, the use of NMDA receptor antagonists reduced the duration of stimulation effects, which probably indicates the key role of synaptic plasticity of glutamatergic neurons in mechanisms of long-term offline effects of tDCS (Liebetanz et al., 2002; Nitsche et al., 2003).

In addition, tDCS can locally reduce the neurotransmission of gamma-aminobutyric acid, regardless of the polarity of the stimulation (Stagg et al., 2009), which can also affect glutamatergic synaptic transmission due to the close relationship between the two neurotransmitter systems. It is also suggested that activation of neurons not only changes their membrane potential and excitation rate, but also reduces electrical resistance. This may be of key importance, since current exposure can cause more significant changes in the transmembrane potential in resting neurons with low membrane conductivity than in active neurons with high membrane conductivity (Paulus and Rothwell, 2016).

In addition to the local effects of tDCS, its effect on the rs-fMRI functional connectivity of the brain has been described. Neural networks react more sensitively to direct current than individual neurons (Francis et al., 2003), and tDCS can change rs-fMRI functional connectivity, synchronization, and oscillatory activity in various cortical and subcortical neuronal networks. This effect has been shown for tDCS of the primary motor cortex (M1) (Polanía et al., 2011a,b, 2012a) and the prefrontal cortex (PFC) (Keeser et al., 2011).

In addition, since tDCS modulates the resting state membrane potential throughout the axons, its use can lead to non-synaptic effects that are likely to affect the persistence of tDCS effects (Ardolino et al., 2005). The non-synaptic mechanisms of tDCS may be based on changes in the conformation and functions of various axonal molecules involved in transmembrane ionic conductivity, axonal transport, as well as changes in the properties of the cell membrane and cytoskeleton (Jefferys, 1995).

Another important fact is that almost all tissues and cells are sensitive to electric fields and, therefore, tDCS can cause changes outside the nervous tissue – in endothelial cells, lymphocytes and glial cells (Ruohonen and Karhu, 2012). This effect, which has not yet been systematically studied, can also contribute to the therapeutic effects of tDCS, since in patients with cerebral diseases, in addition to damage to neurons, other pathological processes (such as neuroinflammation) usually occur.

Due to a possible effect on the inflammatory response, tDCS can theoretically influence the course of the disease (Rabenstein et al., 2019). In addition, DC fields can enhance axon regeneration and neurite growth (Fehlings and Tator, 1992; Wood and Willits, 2006; Pelletier et al., 2014) and, therefore, stimulate the recovery of cognitive functions.

As a result, we can say that tDCS can affect some pathological processes and pathogenetic cascades in the central nervous system, and not just change the excitability of neurons.

### Transcranial Alternating Current Stimulation

Transcranial alternating current stimulation (tACS) is a non-invasive method of stimulating nerve tissue through alternating current of a certain frequency (Paulus, 2011).

**FIGURE 3:**
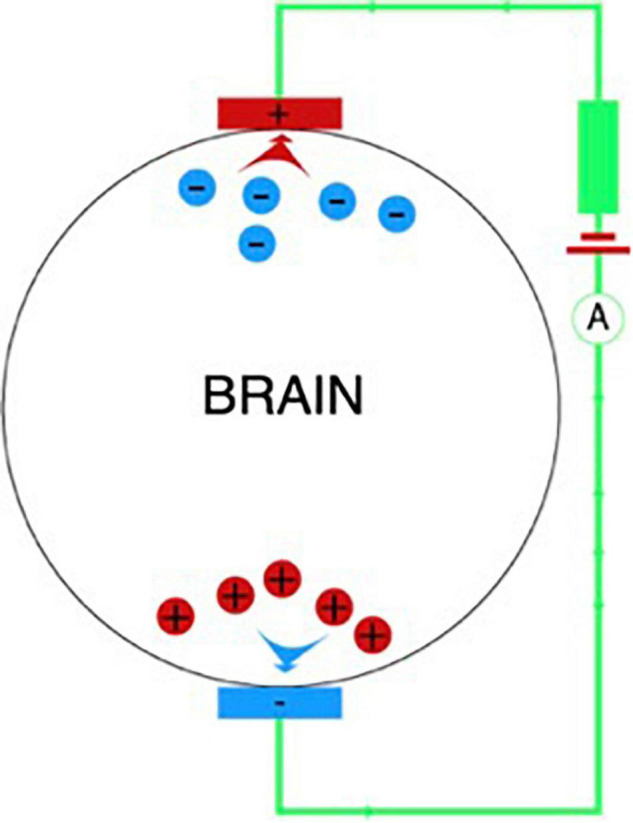
In-phase and antiphase tACS for modulation of the middle frontal theta rhythm.

Unlike tDCS, the advantage of tACS is that it allows to modulate the oscillatory activity of neural networks due to frequency stimulation at an almost imperceptible current strength (Figure 3). Oscillatory activity during exposure is synchronized with external rhythmic stimuli (Herrmann et al., 2013).

During stimulation, the resting potential of the neuron membrane changes at a subthreshold level, which leads to long-term potentiation, in which the suprathreshold peak activity of neurons enhances the connection between neurons and signal propagation through postsynaptic dendrites. This suggests that information on the frequency and phase is the main parameter of the functions of the nervous tissue (Antal and Herrmann, 2016).

Modulation of the oscillatory activity of neural networks by alternating current leads to an “imposition” of exogenous rhythm and an increase in amplitude at an externally specified frequency (Herrmann et al., 2013). To date, it is not clear how long the effects of the imposition of exogenous frequencies after the termination of stimulation can last. One of the mechanisms for preserving the effects of stimulation is changes in neuroplasticity detected after multi-session tACS interventions (Vossen et al., 2015).

An important parameter of tACS is the intensity of the applied alternating current. When stimulating the primary motor cortex with alternating current at a frequency of 140 Hz and simultaneously registering motor evoked potential (MEP) in response to single TMS pulses, it was found that a low stimulation intensity (0.2 mA) leads to inhibition of the cortex, which manifested itself in the form of an increase in the motor response threshold. On the other hand, high intensity (1 mA) leads to lower threshold values, that is, to excitation of the cerebral cortex. The intermediate intensity (0.6 and 0.8 mA) does not affect the motor threshold.

Thus, changes in the excitability of the human cerebral cortex non-linearly depend on the intensity of tACS (Moliadze et al., 2012). This, apparently, indicates that inhibitory neurons are more susceptible to electrical stimulation and are activated even at low current intensities. Excitatory neurons, in contrast, are less susceptible and require more intense stimulation, but dominate inhibitory neurons, which leads to the general excitation effect at high current intensities (Herrmann et al., 2013).

## Transcranial Direct Current Stimulation and Transcranial Alternating Current Stimulation Influence on Cognitive Functions

Neuroplasticity mechanisms are physiological properties that provide reorganization of the nervous tissue. They work by modifying existing neural networks in response to changes in behavior or in the environment (Pascual-Leone et al., 2005; Rossini et al., 2008). It is generally accepted that the same mechanisms are triggered in response to the occurrence of pathological processes in various neurological disorders (Monfils et al., 2005; Kleim and Jones, 2008).

Neuroplasticity is provided by processes such as long-term potentiation (LTP) and long-term depression (LTD), which cause changes in synaptic transmission between neurons (Südhof and Malenka, 2008). These neurophysiological processes significantly affect the functioning of memory and learning mechanisms (Malenka, 1994; Ziemann and Siebner, 2008).

Long-term depression and LTP are controlled and modulated by changing the concentration of dopamine in the basal nuclei (Shen et al., 2008; Schroll et al., 2014). However, in patients with Parkinson’s disease, neuroplasticity processes are disrupted due to pathological processes such as degeneration of dopaminergic neurons and a decrease in dopamine levels in the striatum (Udupa and Chen, 2013; Schroll et al., 2014). This, in turn, can be directly related to memory impairment and a decrease in learning ability (Calabresi et al., 2007; Schroll et al., 2014).

These changes in dopaminergic transmission affect neuroplasticity mainly due to dysregulation between direct and indirect connections in the basal-thalamo-cortical pathways (Figure 1C; Nitsche et al., 2006; Helmich et al., 2009). It has been shown using animal models, that LTP and corticostrial neuroplasticity can be restored by prolonged use of dopamine therapy or transplantation of dopamine neurons (Picconi et al., 2003; Rylander et al., 2013).

Transcranial direct current stimulation has great therapeutic potential because of its modulation of postsynaptic connections effect that is similar to long-term potentiation (Liebetanz et al., 2002). As a result of tDCS, researchers noted an increase in the success rate on tests evaluating the following functions: attention (Elder and Taylor, 2014), semantic (categorical) fluency and overall cognitive function (when combined with physical therapy) (Manenti et al., 2016), executive functions (Doruk et al., 2014), phonetic fluency (Pereira et al., 2012), working memory (Boggio et al., 2006).

Cognitive functions at the neurophysiological level are manifested in the oscillatory activity of neurons, the dynamics of which depend on synaptic activity and membrane potential (Tavakoli and Yun, 2017).

Oscillatory activity of the brain at certain frequencies reflects the activation of specific cognitive or sensorimotor functions (Antal and Paulus, 2013; Herrmann et al., 2013). Thus, tACS can enhance or suppress current processes by exogenously increasing or decreasing the amplitude of oscillations (Herrmann et al., 2013, 2016). Therefore, tACS has the potential to synchronize frequency-specific neural networks, thereby causing changes in behavior (Fröhlich, 2015).

Next, we will examine current views on the effects of tDCS and tACS on cognitive functions such as working memory and cognitive control.

## Working Memory

### Transcranial Direct Current Stimulation

One of the key functions in the cognitive domain is working memory, which provides storage and processing of information for a short period of time. Working memory is associated with various cognitive abilities including selective attention, analysis and decision making (Klingberg, 2010; Jausovec and Jaušovec, 2012; Johnson et al., 2013). The functions of working memory are impaired in various pathological conditions, including Parkinson’s disease (Gilbert et al., 2005).

The nature of working memory deficit in Parkinson’s disease is not yet fully understood. Some researchers (Gabrieli et al., 1996) suggest that dopaminergic dysfunction affects the overall decrease in the rate of psychomotor processes in patients with PD, which causes cognitive impairment, including that associated with working memory.

It is also believed that the working memory deficit in PD is primarily associated with impaired executive functions localized in the dorsolateral prefrontal cortex (DLPFC) (Taylor et al., 1986; Morris et al., 1988; Gabrieli et al., 1996; West et al., 1998). This is explained by a decrease in the dopamine concentration in DLPFC due to degeneration of dopaminergic neurons of the substantia nigra and the ventral region of the tegmentum, disruption of the normal interaction between direct and indirect connections between the cortex, basal nuclei and the thalamus, as well as impaired functioning of neuroplasticity mechanisms (Owen, 2004; Leh et al., 2010). In addition, research results show changes in rs-fMRI functional connectivity in DLPFC in Parkinson’s disease (Wu et al., 2012; Wen et al., 2013; Szewczyk-Krolikowski et al., 2014; Amboni et al., 2015; Trujillo et al., 2015).

In this light, transcranial direct current stimulation of the DLPFC may have a positive effect on working memory deficiency in patients with PD. A possible positive effect of tDCS specific for patients with PD can be the induction of dopamine release into the caudate nucleus via glutamatergic corticostrial pathways, which has been shown in studies on animal models (Whitton, 1997; Strafella et al., 2001; Li et al., 2011; Tanaka et al., 2011; Lu et al., 2015). In addition, it was hypothesized that tDCS in patients with PD can result in neuroprotective effect, which is achieved by reducing oxidative stress in dopaminergic neurons (Lu et al., 2015). Researchers have also found that tDCS modulates rs-fMRI functional connectivity of the cortico-striatal and thalamo-cortical pathways of the human brain (Polanía et al., 2011a).

An analysis of the results presented in the available research on tDCS suggests a positive effect of course stimulation on cognitive function in patients with PD. For example, Boggio et al. (2006) demonstrated the positive effect of anodal tDCS on the working memory of patients with PD. It is worth noting the specificity of the changes, their sensitivity to the brain region and the intensity of stimulation: only the anodal tDCS of the dorsolateral prefrontal cortex was effective by current 2 mA.

Another study evaluated the long-term stimulation effects during the repeated procedure in patients with PD-MCI (Biundo et al., 2015). The authors showed that there was a tendency of working memory functions improvement in the active tDCS group that was observed for 16 weeks. Another study (Lawrence et al., 2018) provided evidence of the effectiveness of cognitive training in combination with tDCS for patients with PD-MCI. The stimulation target for the participants was the left DLPFC (current strength was 1.5 mA), and cognitive training was conducted for 45 min three times a week for 4 weeks. For the active stimulation group, statistically significant improvements were recorded in terms of executive function, attention, working memory, language, daily activity and quality of life. For the same target, in another study (Pereira et al., 2012), one anodal tDCS session increased semantic fluency in patients with PD.

In a placebo-controlled study by Manenti et al. (2016) that used course tDCS of DLPFC in combination with a physiotherapy program, it was shown that such a combination of methods can improve cognitive abilities, fluency of speech, and also increase the results on the PD-CRS (Parkinson’s Disease-Cognitive Rating Scale). It should be noted that the effects persisted for 3 months.

Thus, tDCS of the DLPFC as a method of cognitive deficits correction is a promising approach to adjuvant therapy in patients with Parkinson’s disease and needs further study.

### Transcranial Alternating Current Stimulation

Oscillatory activity of neural networks is probably central to memory processes (Hanslmayr et al., 2019). The functioning of working memory is significantly affected by the coordinated interaction between brain regions mediated by the oscillatory activity of neurons (Roux and Uhlhaas, 2014). In particular, it is assumed that fluctuations in the theta range play a decisive role in organizing patterns of neuronal activity into a serial code, thereby maintaining temporal relationships between objects stored in working memory (Lisman and Jensen, 2013; Roux and Uhlhaas, 2014).

Neural oscillations can organize the information contained in the working memory in time through oscillations in the gamma range (Lisman and Jensen, 2013). In particular, individual gamma cycles can encode individual units of information, and a sequence of objects can then be encoded through several gamma cycles embedded in a theta cycle (Lisman and Idiart, 1995). Moreover, the amplitude of gamma cycles depends on the phase of the theta cycle. Thus, the functioning of such a phase amplitude coupling explains the existence of a hierarchical organization of cortical rhythms (Canolty et al., 2006).

Usually from 4 to 8 gamma cycles are combined into one theta cycle, which allows to encode messages with the corresponding number of elements. Typically, the working memory capacity (also called the range) is seven elements with a standard deviation of up to two elements. It was shown that the range of an individual’s working memory correlates with the number of gamma cycles that fit in one theta cycle (Lisman and Jensen, 2013).

This concept suggests that slowing down the theta rhythm frequency can increase the number of embedded gamma cycles, which, in turn, should increase the working memory bandwidth (Axmacher et al., 2010). Conversely, the acceleration of oscillations in the theta range should reduce the number of gamma cycles and, therefore, reduce the capacity of working memory. This hypothesis has been confirmed by two recent tACS studies, which showed that stimulation at a lower theta frequency increases working memory bandwidth (Vosskuhl et al., 2018; Wolinski et al., 2018).

Wolinski et al. (2018) found that stimulation at higher theta frequencies reduces the working memory bandwidth. Thus, the results of these studies show the existence of a causal relationship between the dynamics of the interaction of theta-gamma-oscillations and a change in the capacity of working memory.

It is assumed that there is an inverse relationship between the frequency of oscillations and the distance between the interacting areas of the brain. Low-frequency oscillations are a fairly global phenomenon that can cover the entire cortex. In contrast, high-frequency oscillations appear to be a local rhythm within a limited cortical region (Tseng et al., 2016). Thus, it is likely that delays in signal propagation between distant regions of the brain are a significant obstacle to the generation and synchronization of oscillatory activity of the brain. It turns out that the shorter the distance, the less the delay in the propagation of action potentials along the axon, and the easier it is to synchronize them at higher frequencies (Fröhlich, 2016).

A recent study on primates showed that theta synchronization between prefrontal and parietal neural ensembles allows more efficient reproduction of information stored in working memory (Jacob et al., 2018). An attempt was also made to test the role of synchronization between the prefrontal and parietal neural networks in the theta range, stimulating the prefrontal and parietal regions inphase (i.e., with zero phase) or antiphase (i.e., 180° from each other).

Both studies showed that working memory performance was improved during synchronization (i.e., inphase stimulation) compared with antiphase stimulation (Polanía et al., 2012b; Violante et al., 2017). Inphase theta stimulation has been found to shorten the response time in the visual memory task, while antiphase stimulation decreases memory performance and increases reaction time (Polanía et al., 2012b). Such a negative effect during antiphase stimulation in the theta range may be explained by the large amount of information contained in each theta cycle compared to the oscillatory cycles of higher frequency ranges, because of which desynchronization during antiphase stimulation leads to a loss of a noticeable amount of information and a decrease in working memory performance (Tseng et al., 2018).

In addition, the desynchronization of the theta phase can lead to impaired integration of distant regions of the brain (Tseng et al., 2018).

## Cognitive Control

Cognitive control is part of the executive function domain and plays a key role in decision-making processes. This term was first used by researchers M. Posner and S. Snyder in their work “Attention and Cognitive Control” (Posner and Snyder, 1975) to define one of the systems responsible for the selection of information, coordination and execution of relevant processes and suppression of irrelevant ones.

The involvement of cognitive control occurs most intensively in situations where there is competition for limited mental resources (Desimone and Duncan, 1995). Its function is to reduce uncertainty in the decision-making process at various levels by regulating the importance of information and its relevance.

For example, during information processing when, in addition to relevant signals, there are factors distracting from the task [as in the Color Stroop (1935) or Eriksen flanker (Eriksen and Hoffman, 1974) tasks], participants must ignore irrelevant information and competing options in order to give an accurate answer. The successful performance in such tasks is based on the use of cognitive control, the function of which is the targeted detection and resolution of conflicts, reduce in uncertainty and facilitation in decision-making (Mackie et al., 2013).

### Transcranial Direct Current Stimulation

According to the conflict monitoring theory (Botvinick et al., 2001), the anterior cingulate cortex (ACC) is involved in detecting conflict and automatically triggering control processes in the DLPFC (Miller and Cohen, 2001; Kerns et al., 2004).

The development of a cognitive control functions deficit in PD is explained by a decrease in the concentration of dopamine in the frontal parts of the cerebral cortex. In addition, it may be due to a decrease in the amplitude of the medial frontal theta rhythm (see the Pathogenesis section) in the anterior cingulate gyrus and, as a result, the cessation of signals from it to the dorsolateral prefrontal cortex.

Based on the literature analysis, it is possible to assume that tDCS of the DLPFC can positively affect cognitive control processes (Reinhart and Woodman, 2014; Zmigrod et al., 2014; Steinhauser et al., 2016). The results of a study by Gbadeyan et al. (2016) using the flanker task, showed the following: cognitive control levels of participants that underwent tDCS over DLPFC were 30% higher compared to the sham group.

Another study by Gbadeyan et al. (2019) presents data showing the effectiveness of tDCS on cognitive control improvement in older people. Regarding the persistence of the effects, there is a study showing the long-term positive effects of tDCS on executive functions of patients with PD (Doruk et al., 2014). In this study course therapy was carried out using anodal tDCS over the right DLPFC.

### Transcranial Alternating Current Stimulation

It is believed that increase in the amplitude of the midfrontal theta rhythm is a marker of the involvement of physiological processes that provide cognitive control (Cavanagh and Frank, 2014). It is also known that its amplitude increases significantly when new stimuli are presented, when solving conflicting problems, and also after making mistakes (Singh et al., 2018).

When solving problems that test the functioning of cognitive control, bursts in the activity of the midfrontal theta rhythm can synchronize the activity of the prefrontal cortex with the underlying structures of the brain, such as the ventral region of the tegmentum, tonsil, septum, and hippocampus. Thus, the medial frontal theta rhythm is likely to be one of the key mechanisms of cognitive control (Cavanagh and Frank, 2014; Singh et al., 2018).

Artificial disfunction of dopamine secretion in rodent models significantly reduces the amplitude of the midfrontal frontal theta rhythm (Parker et al., 2014). Parkinson’s patients also show a decrease in the amplitude of FMT against the background of a deterioration in cognitive control function (Singh et al., 2018) and a decrease in the ability to adapt to new stimuli (Chen et al., 2016; Cavanagh et al., 2017).

## Hypothetical Mechanisms Explaining Positive Effects of Transcranial Brain Stimulation

Much has been said about the importance of physiological rhythms of the brain. In the present review we focused on studies of rhythm and frequency of signals generated during non-invasive stimulation therapy. Mechanisms of the generation of abnormal activity in the basal ganglia, which cause the classic symptoms of PD, e.g., appearance of tremor, rigidity, and bradykinesia, are of interest for research. These mechanisms may explain the short-term and long-term therapeutic effect of non-invasive tDCS therapy. New data indicate that fluctuations in certain frequency ranges that occur in the basal ganglia can be used to diagnose PD. However, not enough is known about the physiological role of such rhythms, the cellular networks that generate them and the functions of such rhythms. It seems plausible that such rhythms can only be an epiphenomenon.

Rhythmic oscillatory activity is present in many different events. The most famous phenomenon associated with oscillators is the hippocampal theta rhythm, the pacemaker of which is the medial septal region (Colgin, 2013). This rhythm is of key importance to the binding of the bursts of impulses of the cell to the place (the phenomenon of phase precession), and is also of great importance in the processes of memory and attention. Generation of spontaneous activity by the brain leads to the appearance of a rhythm of varying amplitude and frequency. Little is known about the role of such spontaneous activity, but it occurs in almost all animals with a nervous system (Hanson, 2021).

Normally, the activity of a neuron is associated with the provision of a signal by it, and depends on a set of input stimuli that it receives. However, the neuron also produces spontaneous activity at the very early stages of the development of the nervous system (Luhmann et al., 2016). Such generators are described, for example, in Marder and Calabrese (1996) Rhythm generators synchronize small neural populations (Ramirez et al., 2004). Other researchers insist that some generators may be fundamental to the whole brain, as such generators can synchronize large populations of neurons (Heck et al., 2017). For humans, the role of Default Mode Network (DMN) in ensuring the operation of generators of various amplitudes is known. The connection between DMN and impaired executive functions was established in PD patients (van Eimeren et al., 2009). When awake, a person produces synchronous rhythmic movements in the form of stereotypical cyclical behavior, such as walking, chewing, breathing, etc. Periodic movements generate the activity of neurons of a certain amplitude and frequency, and vice versa, the frequency of rhythmic activity is associated with the anatomy of movable limbs. de Hemptinne et al. (2013) found that people with Parkinson’s disease have abnormal synchronicity between low-frequency beta waves and high-frequency gamma waves. In particular, the phase of the beta waves was correlated with the amplitude of the gamma waves. They hypothesized that breaking this abnormal synchronicity has a therapeutic effect in the treatment of PD (de Hemptinne et al., 2013) Excessive Phase-amplitude coupling (PAC) leads to degenerate activity of the rhythmic type, which leads to the onset of PD symptoms.

Other studies focus not on rhythm, but on the correlation between the times of spike activity of neurons. Synchronization of spikes on a certain time scale can lead to the appearance of oscillations. Such models insist on pseudo-oscillatory activity of neural networks. It was shown that simultaneous neuronal activity affects neuronal plasticity (Gerstner et al., 1996). Spike-timing-dependent plasticity (STDP) is necessary for the hippocampus to function properly and depends on the frequency and timing of spike synchronization. Synchronized activity of cells innervated by the dopamine system loses its essential properties and gives rise to PD symptoms (Touboul et al., 2020; Bashkirtseva et al., 2021). The PD symptoms in such a model are generated as a result of stochastic resonance, which leads to an increase in periodic oscillations. Due to a decrease in the temporal accuracy of the pulsation of SN cells, which is necessary to maintain the synchronous actuation of the cortical networks, transitions to synchronous states occur at lower frequencies and on a larger scale (Bragin et al., 2002). Accordingly, tACs can synchronize large groups of cortical neurons and lead to amelioration of the PD symptoms.

## Conclusion

Parkinson’s disease manifests itself in both motor and cognitive symptoms. Cognitive deficit significantly affects the quality of life of patients with PD, however, the possibilities of therapy for its correction are significantly lower when compared to therapy of motor manifestations. That is why there is a need in additional methods aimed at correcting cognitive deficits in PD. In this regard, tCS can be potentially effective. The search and adaptation of stimulation protocols, the effectiveness of which will be the highest, requires special attention. Research in this area can not only improve the quality of life of patients with MCI, but also significantly improve understanding of the physiological mechanisms of cognitive impairment in Parkinson’s disease.

Based on the facts presented in this paper, it is possible to suggest the potential effectiveness of MCI correction by stimulating the areas of the brain responsible for these disorders, that is, in particular, the dorsolateral prefrontal cortex (DLPFC), since this area is one of the key anatomical areas for cognitive functions such as working memory and cognitive control. In addition, the prefrontal cortex and, in particular, DLPFC, is involved in compensatory mechanisms in MCI (Gigi et al., 2010) and in reduction of episodic memory in general (Rosano et al., 2012).

On the other hand, the combined evidence of the relationship between FMT and the cognitive domain allows us to hypothesize that it is theoretically possible to modulate the work of cognitive functions impaired in Parkinson’s disease by performing tACS in the theta range. Studies show that the amplitude of the median frontal theta rhythm associated with cognitive control is reduced in Parkinson’s disease. In addition, the theta rhythm is an important mechanism for synchronizing distant regions of the brain and, in particular, is involved in the process of updating the working memory.

## Author Contributions

All authors listed have made a substantial, direct, and intellectual contribution to the work, and approved it for publication.

## Conflict of Interest

The authors declare that the research was conducted in the absence of any commercial or financial relationships that could be construed as a potential conflict of interest.

## Publisher’s Note

All claims expressed in this article are solely those of the authors and do not necessarily represent those of their affiliated organizations, or those of the publisher, the editors and the reviewers. Any product that may be evaluated in this article, or claim that may be made by its manufacturer, is not guaranteed or endorsed by the publisher.
